# New Betulin Derivatives with Nitrogen Heterocyclic Moiety—Synthesis and Anticancer Activity In Vitro

**DOI:** 10.3390/biom12101540

**Published:** 2022-10-21

**Authors:** Ewa Bębenek, Elwira Chrobak, Zuzanna Rzepka, Dorota Wrześniok

**Affiliations:** 1 Department of Organic Chemistry, Faculty of Pharmaceutical Sciences in Sosnowiec, Medical University of Silesia in Katowice, 4 Jagiellońska Str., 41-200 Sosnowiec, Poland; 2 Department of Pharmaceutical Chemistry, Faculty of Pharmaceutical Sciences in Sosnowiec, Medical University of Silesia in Katowice, 4 Jagiellońska Str., 41-200 Sosnowiec, Poland

**Keywords:** pentacyclic triterpenes, heterocyclic derivatives, anticancer activity

## Abstract

As part of the search for new medicinal substances with potential application in oncology, the synthesis of new compounds combining the betulin molecule and the indole system was carried out. The structure of the ester derivatives obtained in the Steglich reaction was confirmed by spectroscopic methods (^1^H and ^13^C NMR, HR-MS). The obtained new 3-indolyl betulin derivatives were evaluated for anticancer activity against several human cancer cell lines (melanomas, breast cancers, colorectal adenocarcinomas, lung cancer) as well as normal human fibroblasts. The significant reduction in MCF-7 cells viability for 28-hydroxy-(lup-20(29)-ene)-3-yl 2-(1*H*-indol-3-yl)acetate was observed at a concentration of 10 µg/mL (17 µM). In addition, cytometric analysis showed that this compound strongly reduces the proliferation rate of breast cancer cells. For this, the derivative showing the promising cytotoxic effect on MCF-7 breast cancer cells, the pharmacokinetic profile prediction was performed using in silico methods. Based on the results obtained in the study, it can be concluded that indole-functionalized triterpene EB367 is a promising starting point for further research in the field of breast cancer therapy or the synthesis of new derivatives.

## 1. Introduction

The global cancer burden is rapidly growing, and it is expected to be about 28.4 million cases in 2040, a 47% rise from 2020. This results from both aging and growth of the population as well as changes in the prevalence of the main risk factors for cancer [1]. The search for new anticancer drugs is the leading direction of scientific research for years. Currently, there is a growing interest in plant-derived compounds in the context of their potential use in oncology [2].

**Figure 1:**
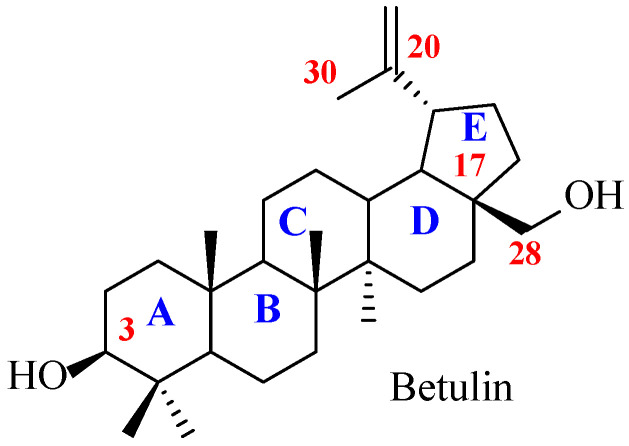
Chemical structure of pentacyclic triterpene-betulin.

Betulin (Figure 1), pentacyclic lupane-structure triterpenoid which occurs abundantly in the birch bark, is one such compound since it has been shown to inhibit growth and migration as well as induce apoptosis of various types of cancer cells. Moreover, betulin can been utilized as a precursor compound for the synthesis of novel derivatives with improved pharmacological properties and potential use in cancer treatment [3,4,5,6,7,8,9,10,11].

**Figure 2:**
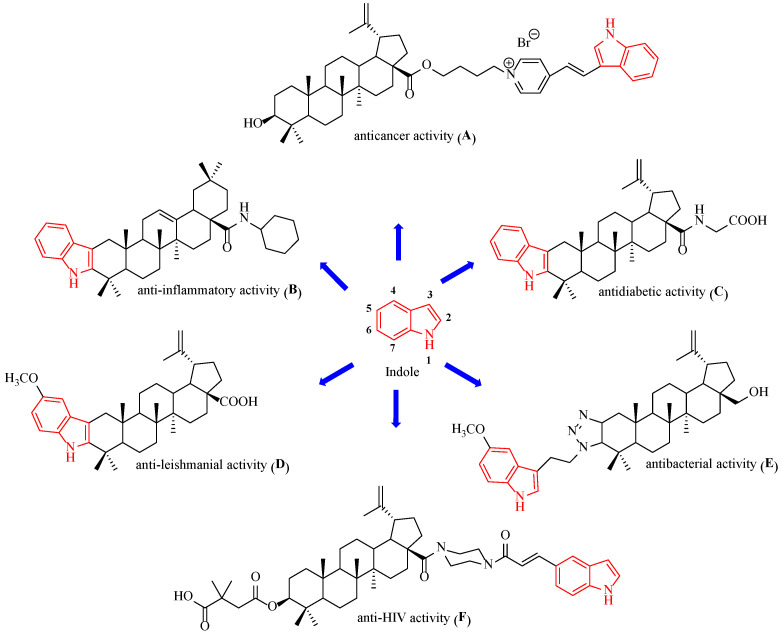
Chemical structures of indole and triterpenes having an indole scaffold.

Indole (1*H*-benzo[b]pyrrole, Figure 2) is a unique chemical structure. It can undergo electrophilic substitution reactions in the benzene ring and nucleophilic substitution reactions on the nitrogen atom in the pyrrole ring under basic conditions due to the acidity of the N-H bond [12].

Indoles represent one of the most important structural classes in drug discovery. Indole nucleus is known to bind to multiple receptors with high affinity and it is found in abundance in biologically active compounds such as alkaloids and pharmaceuticals, including anticancer agents (e.g., vincristine, vinblastine) [13,14]. Indole derivatives have been shown to exert anticancer activity based on various mechanisms such as apoptosis, cell mitosis, cell signal transduction, replication and transcription, and epigenetic modification [12,15].

One method of increasing the biological activity of pentacyclic triterpene derivatives is the modification of the triterpene ring by introducing nitrogen heterocyclic moiety such as the indole system. This made it possible to obtain new compounds with various biological effects, including anticancer, anti-inflammatory, anti-leishmanial, antibacterial, antidiabetic and antiviral properties (Figure 2) [16,17,18,19,20,21,22,23,24,25,26,27].

Mitochondria are organelles present in almost all eukaryotic cells. A characteristic feature of mitochondria is that they differ both in terms of structure and physicochemical properties in normal and neoplastic cells. Their transmembrane potential is higher in neoplastic cells than in normal cells, which enables the accumulation of lipophilic cationic drug molecules in the mitochondria of various tumor cells [16,28]. A well-known mitochondrial-targeting cation is *E*-4-(1*H*-indol-3-ylvinyl)-*N*-methylpyridinium iodide (F16) used in the synthesis of new mitochondrial anticancer agents [29]. The mechanism of action of the F16 is associated with inhibition of the mitochondrial respiratory chain, reduction of intracellular ATP levels, cell cycle arrest and the induction of apoptosis. The action of F16 occurs at the level of the mitochondria in which both necrotic and apoptotic pathways run. The effect of the accumulation of the cationic form F16 in the mitochondria is the depolarization of the membrane and the induction of permeability transition (PT) by influencing proteins that are involved in the control of the permeability of the mitochondrial membrane by direct pore formation or by regulating the opening of the PT pores. These processes disrupt the physiological function of the mitochondria in cancer cells, leading to their death through apoptosis or necrosis [30].

The introduction of the *E*-4-(1*H*-indol-3-ylvinyl)pyridinium moiety into the betulinic acid molecule led to the formation of the F16-conjugated pentacyclic triterpene A (Figure 2). The compound A showed anticancer activity against U937, K562, and Jurkat leukemia cells, with an IC_50_ in the range of 0.616–0.844 µM. In addition, this compound was more cytotoxic than the parent betulinic acid (IC_50_ = 78.540–149.29 µM) against all the tested cancer cell lines [16]. In addition to compound A (Figure 2) consisting of a triterpene system connected to the F16 cationic moiety via butane linker, other the F16-betulinic acid conjugates were synthesized. For these compounds, triethylene glycol was used as a linker. Using rat thymocytes, the role of the mitochondria of the studied cells in the cytotoxic effect of the F16-betulinic acid conjugates was determined. The influence of these hybrid compounds on the major functional parameters of mitochondria was investigated and a model of lecithin liposomes was used to determine their influence on the lipid component of membranes. It has been observed that triterpenoids can affect the opening of the pores of MPT in the mitochondria. Sometimes this process is blocked by a specific cyclosporin A (CsA) inhibitor. The introduction of F16-betulinic acid conjugates into the suspension of liver mitochondria induced swelling of CsA-insensitive organelles. Moreover, the formation of aggregates of the mitochondrium under the influence of F16-betulinic acid conjugates indicates their strong interaction with membranes. The F16-betulinic acid conjugate containing triethylene glycol as a linker changes the surface properties of the mitochondrial and liposomal membranes, inducing permeabilization and aggregation in both models tested. The action of this conjugate may be due to processes such as inhibition of the respiratory chain complexes, protonophore effect and the ability to induce permeabilization of the inner mitochondrial membrane [31].

In the course of research related to the search for new compounds showing mitochondrotoxic properties, hybrid compounds F-16-betulin were obtained. The conjugate of F16 and betulin possessing a butane linker has been shown to have higher antitumor activity compared to the parent F16 cation. The cytotoxic effect of the F16-betulin conjugate is due to its selective accumulation in the mitochondria, which ultimately leads to mitochondrial dysfunction and the overproduction of reactive oxygen species (ROS). The F16-betulin conjugate affects the surface properties of mitochondrial membranes by promoting organelle aggregation through a mechanism insensitive to CsA (MPT pore opening inhibitor) [32].

The objective of this study was to synthesize 3-indole-betulin derivatives. The obtained triterpenes were tested for their cytotoxic and/or cytostatic activity towards several human cancer cell lines (melanomas, breast cancers, colorectal adenocarcinomas, lung cancer) as well as normal human fibroblasts. The compound with greater potential was further analyzed to shed light on the cellular basis of its antitumor action. Additionally, in silico ADMET analysis was performed for the 28-hydroxy-(lup-20(29)-ene)-3-yl 2-(1*H*-indol-3-yl)acetate (EB367) in order to determine their usefulness as therapeutic substance.

## 2. Materials and Methods

### 2.1. General Procedures

Melting points were obtained with an Electrothermal IA 9300 apparatus (Bibby Scientific Limited, Stone, Southampton, GB) at the heating rate of 1 °C/min. Hight-resolution mass spectra (HRMS APCI) were acquired with Bruker Impact II instrument (Bruker). ^1^H and ^13^C NMR spectra of solutions in CDCl_3_ were recorded on the Bruker Avance III 600 spectrometer (Bruker, Billerica, MA, USA). The progress of reactions and the purity of the products were monitored by TLC (silica gel 60 F_254_ plates, Merck, Darmstadt, Germany). The spots were detected by spraying with an ethanolic solution of sulfuric acid and heating to 100 °C. Column chromatography was performed using silica gel 60 (0.063–0.200 mm; Merck, Darmstadt, Germany). Mixture of chloroform and ethanol 40:1 (*v*/*v*) was used as mobile phase. Commercially available reagents were purchased from the Merck (Darmstadt, Germany). The applied solvents were dried according to usual procedures.

### 2.2. Synthesis of 3-Indolyl Betulin Derivatives EB366 and EB367

The staring compounds 1 and 2 were prepared according to the procedures described in the literature [33,34].

#### 2.2.1. General Grocedure for the Steglich Esterification of 28-Tetrahydropyranyl ether of Betulin 1 and 28-Acetylbetulin 2

The solution containing 1.12 mmol DCC and 0.08 mmol DMAP in 1 mL dichloromethane was added dropwise to a mixture of 28-tetrahydropyranyl ether of betulin 1 (or 28-acetylbetulin 2) (1 mmol) and 3-indoleacetic acid (1.10 mmol) in dry dichloromethane (5 mL) in temperature −10 °C. Next, the mixture was stirred at room temperature for 24 h. The reaction completion was monitored by TLC method. At the end, the reaction mixture was filtered to remove a the *N*,*N*′-dicyclohexylurea (by-product). The solvent (dichloromethane) was distilled from the filtrate using a rotary evaporator. The residue was purified by silica gel (SiO_2_) column chromatography with chloroform-ethanol (40:1, *v*/*v*) as an eluent to give compound EB366. The intermediate product 1A was directly employed in the next step of the synthesis, without purification.

#### 2.2.2. General Procedure for the Synthesis of EB367

The intermediate product 1A (0.38 mmol) was dissolved in ethanol (13 mL) and then pyridinium p-toluenesulfonate (PPTS) (0.71 mmol) was added. The reaction was stirred at room temperature for one week. Next, ethanol was distilled off using a rotary evaporator. The obtained residue was dissolved in dichloromethane (5 mL), washed with a saturated sodium bicarbonate solution (4 mL) and dried with anhydrous sodium sulfate. Dichloromethane was distilled off using a rotary evaporator. The crude product EB366 was purified by column chromatography (SiO_2_, chloroform-ethanol, 40: 1, *v*/*v*).

28-Acetyl-lup-20(29)-ene-3-yl 2-(1*H*-indol-3-yl)acetate EB366 Yield 48%; m.p. 124–126 °C; R_f_ = 0.51 (chloroform/ethanol, 40:1, *v*/*v*);^1^H NMR (CDCl_3_), 600 MHz) δ 0.78 (s, 3H, CH_3_), 0.80 (s, 3H, CH_3_), 0.85 (s, 3H, CH_3_), 0.98 (s, 3H, CH_3_), 1.04 (s, 3H, CH_3_), 1.70 (s, 3H, CH_3_), 2.09 (s, 3H, CH_3_C = O), 2.46 (m, 1H, H-19), 3.79 (s, 2H, CH_2_C = O), 3.86 (d, 1H, *J* = 11.4 Hz, H-28), 4.26 (d, 1H, *J* = 10.8Hz, H-28), 4.51, (m, 1H, H-3), 4.60 (s, 1H, H-29), 4.70 (s, 1H, H-29), 7.14–7.65 (m, 5H, indole), 8.08 (s, 1H, N-H, indole) (Figure S1 Supplementary Materials); ^13^C NMR (CDCl_3,_ 150 MHz) δ 14.74, 16.03, 16.15, 16.50, 18.12, 19.10, 20.79, 21.09, 23.66, 25.13, 27.04, 27.85, 29.56, 29.73, 31.72, 34.10, 34.55, 37.05, 37.55, 38.35, 40.88, 42.69, 46.30, 47.72, 48.76, 50.25, 55.36, 62.83, 81.31, 109.01, 109.90, 111.01, 111.06, 119.00, 119.58, 122.16, 122.87, 127.31, 136.06, 150.17, 171.69, 171.88 (Figure S2 Supplementary Materials). HRMS (APCI) *m/z*: calcd for for C_42_H_58_NO_4_ [(M-H)^−^]: 640.4366; found 640.4364 (Figure S3 Supplementary Materials).

28-Hydroxy-(lup-20(29)-ene)-3-yl 2-(1*H*-indol-3-yl)acetate EB367 Yield 52%; m.p. 99–101 °C; R_f_ = 0.32 (chloroform/ethanol, 40:1, *v*/*v*); ^1^H NMR (CDCl_3,_ 600 MHz) δ 0.78 (s, 3H, CH_3_), 0.80 (s, 3H, CH_3_), 0.85 (s, 3H, CH_3_), 0.98 (s, 3H, CH_3_), 1.07 (s, 3H, CH_3_), 1.70 (s, 3H, CH_3_), 2.40 (m, 1H, H-19), 3.34 (d, 1H, *J =* 10.8 Hz, H-28), 3.80 (m, 3H, H-28, O = CCH_2_), 4.51 (m, 1H, H-3), 4.60 (s, 1H, H-29), 4.69 (s, 1H, H-29), 7.14–7.65 (m, 5H, indole), 8.08 (s, 1H, N-H) (Figure S4 Supplementary Materials); ^13^C NMR (CDCl_3,_ 150 MHz) δ 14.73, 15.69, 16.16, 16.50, 18.13, 19.05, 20.82, 23.65, 25.13, 27.01, 27.85, 29.14, 29.71, 31.72, 33.96, 34.13, 37.84, 38.33, 40.91, 42.70, 47.78, 47.81, 48.72, 50.26, 55.35, 60.56, 81.31, 109.01, 109.75, 111.02, 111.07, 119.00, 119.58, 122.16, 122.72, 122.88, 127.31, 136.05, 150.48, 171.89 (Figure S5 Supplementary Materials). HRMS (APCI) *m/z*: calcd for C_40_H_57_NO_3_ [(M-H)^−^]: 598.4260; found 598.4274 (Figure S6 Supplementary Materials).

### 2.3. Cell Culture

Human cancer cell lines: A375 (CRL-1619), C32 (CRL-1585), MDA-MB-231 (HTB-26), MCF-7 (HTB-22), DLD-1 (CCL-221), HT-29 (HTB-38), A549 (CCL-185) were purchased from ATCC (Manassas, VA, USA). Normal human fibroblasts were obtained from Sigma Aldrich Inc. (St. Louis, MO, USA). HT-29 cells were cultured in McCoy’s 5a medium (Sigma Aldrich Inc., St. Louis, MO, USA), DLD-1 cells were cultured in RPMI 1640 medium (PAN-Biotech, Aidenbach, Germany), and normal human fibroblasts were cultured in Fibroblast Growth Medium, all-in-one ready-to-use (Sigma Aldrich Inc., St. Louis, MO, USA). The remaining cell lines were cultured in Dulbecco’s Modified Eagle’s Medium (Thermo Fisher Scientific, Waltham, MA, USA). Culture media, except from the one for normal fibroblasts, were supplemented with 10% (*v*/*v*) fetal bovine serum (Gibco, Waltham, MA, USA) as well as penicillin (100 U/mL), neomycin (10 µg/mL) and amphotericin B (0.25 µg/mL) obtained from Sigma Aldrich Inc. (St. Louis, MO, USA). Cells were maintained at 37 °C in a CO_2_ incubator CB 160 (BINDER, Tuttlingen, Germany) in humidified atmosphere with 5% CO_2_. The novel betulin derivatives EB366 and EB367 were dissolved in dimethyl sulfoxide (Sigma Aldrich Inc., St. Louis, MO, USA) to prepare 10 mg/mL stock solutions, which were used to prepare dilutions in culture media.

### 2.4. WST-1 Assay

The WST-1 test (Roche GmbH, Mannheim, Germany) was used to explore the metabolic activity of the mitochondria and thus the vital status of cells. Cells in the number of 2.5 × 10^3^ cells per well were seeded into 96-well microplates and incubated overnight. Next, the cells were treated with the novel betulin derivatives in concentrations ranging from 1 to 100 µg/mL or with 1% dimethyl sulfoxide (control). After 70 h of the treatment, 10 µL of WST-1 reagent was added to each well, and the incubation was continued for 2 h. The absorbance measurement was made using microplate reader Infinite 200 PRO (TECAN, Männedorf, Switzerland) at 440 nm and 650 nm as a reference wavelength. Control samples were normalized to 100%, and all tested samples were calculated as the percentage of the control.

### 2.5. Fluorescent Cytometry Analysis

Nucleocounter NC-3000 (ChemoMetec, Lillerød, Denmark) controlled by the NucleoView NC-3000 Software was applied to assess cell proliferation rate, cell membrane damage and DNA fragmentation.

Cell count and viability assay is based on the staining of non-fixed cells with acridine orange (AO) and DAPI. AO is a membrane-permeable dye that binds to nucleic acids, staining all the cells in a population. DAPI cannot penetrate the cell membrane, hence it only stains cells with a damaged cell membrane (dead cells). After the treatment, cells were trypsynized, centrifuged and resuspended in the culture medium. Next, cell suspension was loaded into cassette containing the stains and immediately analyzed by the NC-3000 system. 

DNA fragmentation was evaluated following a previously described method [35]. In brief, following the treatment, cells were fixed with ice-cold 70% ethanol at 4 °C for 24 h, stained with Solution 3 (ChemoMetec, Lillerød, Denmark) for 5 min at 37 °C, and then analyzed using the fluorescent cytometer.

### 2.6. Microscopic Assessment of Cell Culture

Cells were grown on sterile cover slips placed in Petri dishes. After treatment, cells were fixed (4% paraformaldehyde), permeabilized (0.1% Triton X-100) and stained with 0.6 µM Phalloidin–Atto 565 (Sigma Aldrich Inc., St. Louis, MO, USA) and 1 µM SYTO Deep Red Nucleic Acid Stain (Thermo Fisher Scientific, Waltham, MA, USA). The samples were scanned using the laser confocal microscope Nikon Eclipse Ti-E A1R-Si and Nikon NIS Elements AR software.

### 2.7. In Silico Analysis

Freely accessible web tolls were used to analyze and predict the pharmacokinetic profile of EB367. Some molecular and physicochemical descriptors as well as drug likeness were evaluated using the SwissADME (SIB Swiss Institute of Bioinformatics, Molecular Modeling Group, Quartier Sorge, Bâtiment Génopode, Lausanne, CH-1015, Switzerland, http://www.swissadme.ch, accessed on 14 August 2022) [36]. Target identification was performed by SwissTargetPrediction (SIB Swiss Institute of Bioinformatics, Molecular Modeling Group, Quartier Sorge, Bâtiment Génopode, Lausanne, CH-1015, Switzerland, http://www.swisstargetprediction.ch, accessed on 20 August 2022) [37]. The pharmacokinetic (ADME) profile of derivative was predicted using the admetSAR version 2.0 server (East China University of Science and Technology, School of Pharmacy, Shanghai Key Laboratory of New Drug Design, Laboratory of Molecular Modeling and Design http://lmmd.ecust.edu.cn/admetsar2, accessed on 24 June 2022) [38]. The toxicity studies were performed by ProTox-II (Charite University of Medicine, Institute for Physiology, Structural Bioinformatics Group, Philippstrasse 12, 10115 Berlin, Germany https://tox-new.charite.de, accessed on 23 August 2022) [39].

### 2.8. Statistical Analysis

Data were analyzed using GraphPad Prism 8 (GraphPad Software, San Diego, CA, USA). Statistical significance of differences was tested by one-way ANOVA with Dunnet’s post hoc test based on the results of three independent experiments. A *p*-value of < 0.05 was considered indicative of a statistically significant difference.

## 3. Results and Discussions

### 3.1. Chemistry

The tetrahydropyranyl (THP) moiety protects the hydroxyl groups in alcohols used as substrates or intermediates in multistage organic synthesis. THP ethers are attractive compounds due to the low cost of dihydropyran (DHP) used in their preparation. The THP-derivatives are stable under various reaction conditions. Moreover, the THP protecting group can be easily removed under slightly acidic conditions. The advantage of the THP group over the benzyl-based protecting groups is associated with the obtaining of protective compounds with better solubility [40].

**Scheme 1:**
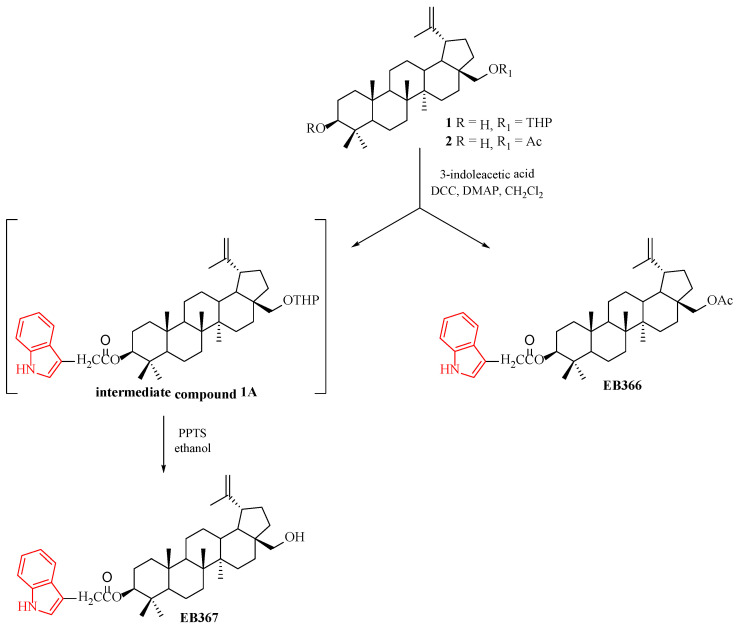
Synthetic route for the preparation of 3-indolyl derivatives of betulin.

In the synthesis of tetrahydropyranyl ether 1 was applied a mild and efficient method by the THP protection of the primary hydroxyl group at the C-28 position of betulin [33]. The esterification reaction of the secondary hydroxyl group at the C-3 position of compound 1 by using 3-indoleacetic acid produced intermediate product 1A. The 3-indole-betulin derivative EB367 was obtained by the deprotection of the THP group of intermediate compound 1A by treatment of pyridinium p-toluenesulfonate (PPTS) in ethanol. The derivative EB366 was obtained in the Steglich esterification between 28-acetylbetulin 2 and 3-indoleacetic acid in the presence of *N,N*′-dicyclohexylcarbodiimide (DCC) and 4-dimethylaminopyridine (DMAP). The compounds EB366 and EB367 were obtained in 48–52% yield. The chemical structures of new 3-indolyl substituted derivatives of betulin EB366 and EB367 were confirmed by ^1^H NMR, ^13^C NMR, and HRMS techniques (Supplementary Materials, Figures S1–S6). The synthetic route for the preparation of 3-indolyl derivatives of EB366 and EB367 is shown in Scheme 1.

### 3.2. Screening Cancer Cell Lines for Sensitivity to Novel Betulin Derivatives

**Figure 3:**
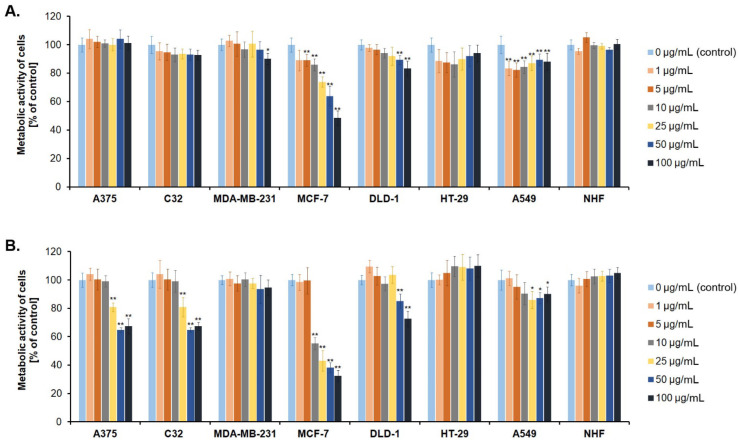
The effect of novel betulin derivatives EB366 (**A**) and EB367 (**B**) on metabolic activity of human cancer cell lines: A375, C32, MDA-MB-231, MCF-7, DLD-1, HT-29, A549 and normal human fibroblasts (NHF). The cells were analyzed using WST-1 assay following 72 h treatment with the derivatives at the concentration ranging from 1 to 100 µg/mL. The results were expressed as a percentage of the mean value obtained for control (the cells treated with 1% DMSO). Bars represent mean values ± standard deviation calculated from three independent experiments. * *p* < 0.05, ** *p* < 0.01 vs. control.

We investigated if the treatment with EB366 and EB367 affects the number of metabolically active cells in experimental models of various type of cancer. WST-1 assay was performed for human melanoma cell lines (A375, C32), human breast cancer cell lines (MDA-MB-231 and MCF-7), human colorectal adenocarcinoma cell lines (DLD-1 and HT-29) and human lung carcinoma A549 cells. In parallel, the analysis was carried out on normal human fibroblasts (NHF). The results are shown in Figure 3 and the data from IC_50_ estimation are presented in Table S1 (Supplementary Materials).

As shown in Figure 3, the triterpene EB366 had a selective effect on the MCF-7 cell line. The compound reduced the viability (metabolic activity) of MCF-7 human breast cancer cells in a concentration-dependent manner. The concentration of EB366 required to reduce MCF-7 cells survival by 50% (IC_50_) was 156 µM (100 µg/mL). In this concentration, EB366 did not affect viability of normal human fibroblasts. The second tested derivative, EB367, has been shown to be more effective against MCF-7 cells than EB366, since IC_50_ was estimated to be 35 µM (21 µg/mL). In our previous study [41], we determined the IC_50_ value of cisplatin, the reference drug. The estimated value for MCF-7 cells was 5.5 µM, which is lower than the value demonstrated here in the case of EB367. Nevertheless, as we have shown previously [42], cisplatin exhibits high cytotoxicity towards normal fibroblasts (IC_50_ = 9.1 µM) in contrast to EB367, which displays the selectivity of action against MCF-7 breast cancer cells. The compound EB367 in a concentration 50 µg/mL (83 µM) and 100 µg/mL (167 µM) caused a decrease in a cellular viability by about 30–35% in both tested melanoma cell lines (C32 and A375). The similar effect was also observed in the case of colorectal adenocarcinoma DLD-1 cells treated with EB367 in a concentration of 100 µg/mL. Importantly, no cytotoxic effect of the compound was noted on normal human fibroblasts.

It is well known that cancer cells, to achieve rapid proliferation, modify their energy metabolism, mainly by preferring aerobic glycolysis or enhancing the oxidative phosphorylation (OXPHOS) [43,44]. Metabolic heterogeneity in breast cancer cell lines has been demonstrated as an important cause of resistance to anticancer agents. MDA-MB-231 cells, the triple-negative breast cancer (TNBC) cell line, when compared to MCF-7 cells (luminal A breast cancer subtype cell line) display lower OXPHOS activity due to molecular alteration such as functional defects in some respiratory complexes and mTOR pathway. Thus, MDA-MB-231 cells are characterized by the predominance of glycolysis, whereas MCF-7 cells produce ATP mainly through OXPHOS [45]. In our study, we noted a significant difference between MCF-7 and MDA-MB-231 cell line in the response to the treatment with EB366 and EB367. This phenomenon may be due to the ability of the studied derivatives to induce metabolic stress. Reda et al. [45] have revealed that inhibition of glycolysis in MDA-MB-231 and MCF-7 cells, induces apoptosis only in the latter cell line, whereas TNBC cells survive as a result of the activation of mitochondria.

### 3.3. Analysis of Proliferation Rate and Survival of Breast Cancer Cells Treated with EB367

In the next step of our in vitro study, we investigated whether the significant reduction of viable cells observed for MCF-7 cell line was due to inhibition of cell division and/or induction of cell death. The cells were incubated in medium (control) or treated with the compound at the concentration of 25 and 50 µg/mL for 72 h and then analyzed using a fluorescent cytometer. We observed, that the triterpene EB367 inhibited MCF-7 cells proliferation by approx. 55–57% in comparison to the control (Table 1). Dead cells are characterized by a damaged cell membrane, and the hallmark of apoptosis is DNA fragmentation [46]. Comparing the population of cells treated with the compound EB367 and control cells, there were no significant changes in the percentage of cells with a damaged cell membrane or fragmented DNA. Therefore, cytostatic but not cytotoxic activity towards MCF-7 cells was demonstrated for the compound EB367 in both tested concentrations.

**Figure 4:**
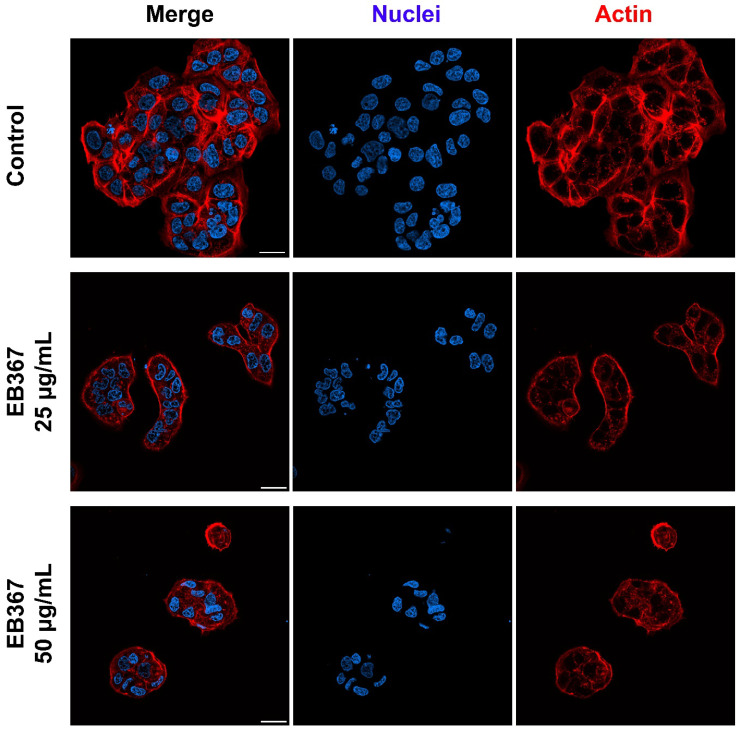
Representative confocal microphotographs of MCF-7 cells treated with the new triterpene EB367. The cells were stained with SYTO Deep Red Nucleic Acid Stain and Phalloidin–Atto 565 to visualize nuclei and actin filaments, respectively. Scale bar shows 25 µm.

The anti-proliferative effect of EB367 against MCF-7 cells was also examined using confocal microscope. The obtained representative images are shown in Figure 4. When compared to control, the population of cells treated with EB367 in both tested concentrations demonstrated a significant reduction of confluency and loss of intercellular contact. As presented in Figure 4, treated cells grew in few-cell groups instead of extensive clusters observed in the control sample.

According to the latest report published by the International Agency for Research on Cancer (the cancer agency of the World Health Organization), cancer ranks first or second leading cause of premature death in most countries. The most commonly diagnosed malignancies worldwide are female breast cancer (incidence: 2.1 million new cases in 2018; mortality: 627,000 deaths in 2018) and lung cancer (incidence: 2.1 million new cases in 2018; mortality: 1.8 million deaths in 2018) [47]. There is still an urgent need for development of novel therapeutic approaches to lower the mortality and morbidity originated from oncological diseases.

Plant-derived compounds have been an important source of several clinically useful anticancer agents, such as vinblastine, vincristine, topotecan, irinotecan, etoposide and paclitaxel [48]. In many in vitro studies, betulin and betulinic acid have been shown to possess a wide spectrum of pharmacological effects, including anti-inflammatory, antimicrobial and anticancer. However poor water solubility of betulin limits its efficacy and potential as a pharmaceutical agent [49]. The structure of betulin enables chemical modifications and therefore in it has been used as a precursor for the synthesis of novel derivatives with improved pharmacokinetic and pharmacodynamic properties. For example, previous studies indicated, that carbamate and N-acylheterocyclic bearing derivatives of betulin showed selective cytotoxic activity against various cancer cell lines [50,51]. Indole ring is considered one of the most privileged scaffolds in drug development [13]. Therefore, in the present study, we synthesized new 3-indole-betulin derivatives.

Promising results of screening for anticancer activity of derivative EB366 were obtained on the estrogen-receptor-positive breast cancer cells. In the case of derivative EB367 a significant decrease in the metabolic activity was also observed for C32 and A375 melanoma cells as well as for DLD-1 colon cancer cell line. The same as the compound EB366, the derivative EB367 had the most prominent effect on MCF-7 cells. Our cytometric analysis and microscopic observations revealed, that the triterpene EB367 in a concentration of 25 µg/mL strongly reduced the proliferation rate of breast cancer cells.

Based on the results of this study, one may conclude, that the presence of free hydroxyl group at the C-28 position of the indole-functionalized derivatives of betulin was advantageous for anticancer activity. In our previous study [41] it was demonstrated that betulin in a concentration ranging from 0.01 to 100 µM did not affect the viability of MCF-7 cells. Therefore, adding an indole group at the C-3 position of betulin allows to obtain derivatives with enhanced anticancer activity against estrogen-receptor-positive breast tumor cells. Previously, breast cancer cells have been shown to be sensitive to certain betulin derivatives, that contain a 1,2,3-triazole ring [41] or sulfonamide group [52].

Selectivity towards cancer cells is an important aspect when developing compounds with potential use in oncology. Therefore, in our study of cell viability, we included human normal fibroblasts, and the obtained results indicate the absence of cytotoxicity of the novel derivatives towards normal cells.

### 3.4. In Silico

At the various stages of discovery and development of new drug substances, it is very important to understand the metabolism and pharmacokinetics of a new drug candidate. The selected chemical compound should have a balance between appropriate potency, pharmacokinetic properties, ADME parameters (Absorption, Distribution, Metabolism, Excretion) and a safety profile ensuring an appropriate dose with a minimum level of drug interaction and adverse side effects. In the process of developing new therapies for various human diseases, researchers seek to discover and develop better drugs in less time and at less cost [53]. For this purpose, in the study of the properties of drug candidates, new tools for obtaining information, i.e., in silico techniques, were introduced [54].

Taking into account the promising cytotoxic effect of the compound EB367 on MCF-7 breast cancer cells and its significant selectivity, an important element of further research was the analysis of pharmacokinetic parameters and the assessment of its drug-like nature. The prediction of these characteristics was carried out using a free online tool (SwissADME). Table 2 presents the most important physicochemical parameters of compound EB367.

The effectiveness of the drug substance results, inter alia, from its bioavailability related to the structure and physicochemical parameters. There are several rules for determining the drug-likeness of a chemical substances based on the value requirements of these descriptors [55]. The first and still commonly used is the “rule of five” formulated by Lipinski, which defines a compound with good oral absorption. Its basic guidelines are the values of such parameters as log *p* < 5; MW < 500 Da; H-BD < 5 and H-BA < 10 [56]. The derivative EB367 we are examining violates two Lipinski criteria (Supplementary Materials, Table S2) which are the molar mass (MV > 500) and lipophilicity (MLOGP > 4.15). The most violations, as many as four, were found against the Ghose rule (MW > 480, WLOGP > 5.6, MR > 130, number of atoms > 70) [57]. The rules formulated by Egan and Muegge were exceeded only in terms of lipophilicity [58,59]. The “rule of five” applies to orally administered drugs, while the rules developed by Veber taking into account only two parameters such as the number of rotational bonds (RB < 10) and topological polar surface of molecule (TPSA < 140 Å^2^) apply to substances administered by routes other than oral [60]. Examined compound follows the Veber’s rule (Supplementary Materials, Table S2).

Despite the fact that the Lipinski rule meets most of the orally used drugs, its stringent requirements regarding physicochemical properties may mean that a promising drug candidate may be rejected at a too early stage of research [61,62].

Compounds characterized by a high proportion of sp^3^ (Fsp^3^) centers have been found to be promising drug candidates. Such structures are similar to natural compounds, are more three-dimensional (3D) and offer the possibility of further advances in the design of complex molecules. Increasing the character of sp^3^ is associated with the presence of substituents outside the plane, which can lead to a better adaptation of the shape of the molecule to the molecular target. The generation of interactions inaccessible to a planar ligand system in this way can increase the strength and selectivity of the molecule to a given target. The Fsp^3^ value calculated for the compound EB367 is consistent with the required (1 > Fsp^3^ > 0.25) and is equal to 0.72 [63].

**Figure 5:**
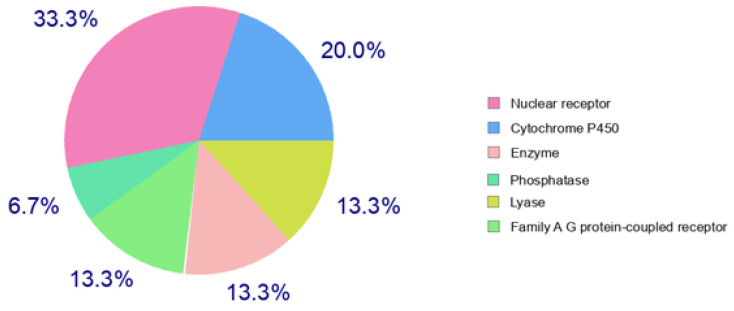
Summary of biological target classes for EB367 predicted using the SwissTargetPrediction web tool.

Further in silico studies aimed to determine to which class the potential therapeutic targets for EB367 will belong. The classes selected using the SwissTargetPrediction platform and their percentage (calculated using the top 15) are presented in Figure 5.

The largest share was predicted for the nuclear receptor (NR) superfamily (33.3%) and the cytochrome P450 family (20.0%). Nuclear receptors are a superfamily of transcription factors that are important therapeutic targets. They control a variety of physiological processes including metabolism, homeostasis, differentiation, growth and development, aging, and reproduction [64]. Among the enzymes of the cytochrome P450 family, the highest probability was predicted for the interaction of EB367 with isoforms CYP17A1 (related to endocrine effects and metabolism of steroid hormones) and CYP19A1 (related to androgenic precursors transformation into estrogens) [65].

For a more complete characterization (Supplementary Materials, Table S3) of the tested compound, it was necessary to determine its pharmacokinetic profile. Predicting ADME parameters was carried out using the admetSAR version 2.0 server (Supplementary Materials, Table S4). Computational analysis showed that EB367 undergoes human intestinal absorption (HIA) but does not show Caco-2 permeability. The parameters related to the absorption of the drug substance are related to the physicochemical properties of the molecule, including lipophilicity, the high value of which affects the predicted poor oral bioavailability.

In silico analysis of EB367 distribution predicted mitochondria as its subcellular localization. As predicted, EB367 is characterized by crossing the blood-brain barrier. High lipophilicity of this compound may facilitate the process of overcoming this barrier, but it is a complex process [62]. Currently, in addition to the traditional approach (passive and active transport) [66], some authors suggest that all compounds cross membranes with the participation of transmembrane proteins [67].

As evidenced by in silico predictions, the test compound may potentially be a substrate of P-glycoprotein (Pgp-ABC-type transporting protein), but also its inhibitor. Overexpression of Pgp in cancer cells can hinder drug delivery and cancer targeting. The ability to inhibit this protein can be used to increase the effectiveness of anti-cancer drugs [68].

Similarly, the interaction of the compound EB367 with the cytochrome P450 3A4 isoform for which it can be both a substrate and an inhibitor was predicted. Cytochrome P450 is responsible for the metabolism and catalyzes the oxidation of endogenous substances and xenobiotics, including approx. 70% of the most commonly used drugs [69] that the compound EB367 may be an inhibitor of organic anion transporting polypeptides OATP1B1 and OATP1B3. Organic anion transporting polypeptides are among the major transporters in the distribution of drugs and other xenobiotics. Alteration of the activity of OATP proteins, including OATP1B1, OATP1A2, OATP1B3 and OATP2B1, may affect the pharmacokinetics and then the effectiveness of some drugs [70].

The action of the drug substance is a complex process which, in addition to the expected effect, also causes a number of side effects. The process of designing new drugs should take into account not only the biological goals associated with the treatment of a given disease, but also other goals that can be predicted in order to eliminate potential negative effects on the organism. The results of the prediction of various toxicity endpoints (acute toxicity, hepatotoxicity, cytotoxicity, carcinogenicity, mutagenicity, immunotoxicity) adverse outcomes pathways (Tox21) and toxicity targets, obtained by in silico method, are presented in Table 3.

In the literature on pentacyclic triterpenes, the low toxicity of betulin and betulinic acid, which has been repeatedly confirmed in studies, is often emphasized [72,73,74]. The in silico results for the EB367 derivative showed that the introduction of an ester-linked indole system into the betulin scaffold should not result in increased toxicity. The EB367 is reported with a toxicity class 5 (according to the Globally Harmonized System of classification of labelling of chemicals-GHS) with LD50 value of 5000 mg/kg, with an average similarity of 57.32% and prediction accuracy of 67.38%. The toxicological endpoint of EB367 (immunotoxicity) is predicted with a confidence score of 0.89.

## 4. Conclusions

The resulting new 3-indolyl substituted betulin derivatives were assessed for anti-tumor activity against human cancer cell lines such as A375, C32, MDA-MB-231, MCF-7, DLD-1, HT-29 and A549. In the conducted studies, a difference was noticed between the two breast cancer cell lines used (MCF-7, MDA-MB-231) in response to treatment of tested compounds. It can be assumed that this phenomenon is related to the ability of the 3-indolyl substituted betulins to induce metabolic stress. 28-Hydroxy-(lup-20(29)-ene)-3-yl 2-(1*H*-indol-3-yl) acetate (EB367) containing a free hydroxyl group at the C-28 position showed significant antitumor activity against MCF-7 cells. Due to the high selectivity of the compound EB367 on MCF-7 breast cancer cells, the analysis of its pharmacokinetic parameters was performed. In silico analysis for compound EB367 indicates that introducing into the structure of betulin at the C-3 position of the ester-linked indole system should not increase toxicity.

In conclusion, findings from our in vitro study suggest that indole-functionalized triterpene EB367 may be considered as a potential anti-breast cancer agent for further analysis as well as used as a skeleton structure for developing new chemotherapeutics.

## Figures and Tables

**Table 1:** The effect of EB367 on proliferation rate (% of control), cell membrane damage and DNA fragmentation of MCF-7 cells in vitro. Data presented as mean ± standard deviation (SD) of 3 independent experiments.

Concentration of EB367	Relative Cell Number [Mean Value ± SD]	Percentage of Cells with Damaged Cell Membrane [Mean Value ± SD]	Percentage of Cells with Fragmented DNA [Mean Value ± SD]
0 µg/mL (control)	100 ± 7	3.9 ± 1.9	1.3 ± 0.6
25 µg/mL	45 ± 9 **	5.0 ± 1.0	2.7 ± 1.2
50 µg/mL	43 ± 6 **	7.8 ± 1.6 *	3.1 ± 0.9

* *p* < 0.05, ** *p* < 0.01 vs. control.

**Table 2:** Selected physicochemical properties of EB367 (according to the SwissADME).

Parameter	Value
Formula	C_40_H_57_NO_3_
Molecular weight (MW)	599.89 g/mol
Fraction Csp^3^ (Fsp^3^)	0.72
Number of rotatable bonds (RB)	6
Number of H-bond acceptors (H-BA)	3
Number of H-bond donors (H-BD)	2
Molar Refractivity (MR)	182.38
Topological polar surface (TPSA)	62.32 Å²
Log *P*_o/w_ (WLOGP)	9.27
Log *P*_o/w_ (XLOGP3)	10.58
Log *P*_o/w_ (MLOGP)	6.61

LogP—log octanol/water partition coefficient calculated by different algorithms.

**Table 3:** Toxicity report predicted by Pro Tox-II web-server for EB367.

Classification	Target	Prediction	Probability
Organ toxicity	Hepatotoxicity	Inactive	0.65
Toxicity end points	Carcinogenicity	Inactive	0.62
	Immunotoxicity	Active	0.89
	Mutagenicity	Inactive	0.72
	Cytotoxicity	Inactive	0.78
Tox21-Nuclear receptor signaling pathways	Aryl hydrocarbon Receptor (AhR)	Inactive	0.84
	Androgen Receptor (AR)	Inactive	0.84
	Androgen Receptor Ligand Binding Domain (AR-LBD)	Inactive	0.78
	Aromatase	Inactive	0.82
	Estrogen Receptor Alpha (ER)	Inactive	0.73
	Estrogen Receptor Ligand Binding Domain (ER-LBD)	Inactive	0.88
	Peroxisome Proliferator Activated Receptor Gamma (PPAR-Gamma)	Inactive	0.92
Tox21-Stress response pathways	Nuclear factor (erythroid-derived 2)-like 2/antioxidant responsive element (nrf2/ARE)	Inactive	0.88
	Heat shock factor response element (HSE)	Inactive	0.88
	Mitochondrial Membrane Potential (MMP)	Inactive	0.68
	Phosphoprotein (Tumor Supressor) p53	Inactive	0.79
	ATPase family AAA domain-containing protein 5 (ATAD5)	Inactive	0.94

(Tox21) Toxicology in the 21st Century program [71].

## Data Availability

Not applicable.

## Supplementary Materials

The following supporting information can be downloaded at: https://www.mdpi.com/article/10.3390/biom12101540/s1, Figure S1: ^1^H-NMR spectrum for EB366 (600 MHz, CDCl_3_); Figure S2: ^13^C-NMR spectrum for EB366 (150 MHz, CDCl_3_); Figure S3: HRMS spectrum for EB366; Figure S4: ^1^H-NMR spectrum for EB367 (600 MHz, CDCl_3_); Figure S5: ^13^C-NMR spectrum for EB367 (150 MHz, CDCl_3_); Figure S6: HRMS spectrum for EB367. Table S1. Cytotoxic in vitro activity of indole-functionalized derivatives of betulin, expressed as IC_50_ values; Table S2. Drug-likeness of EB367 predicted using the SwissADME; Table S3. The in silico predictions of human proteins most likely interacting with EB367 computed using SwissTargetPrediction tool; Table S4. Prediction of the ADME profile of compound EB367 based on computer calculations using admetSAR.
