# Supplementary material for: New Betulin Derivatives with Nitrogen Heterocyclic Moiety—Synthesis and Anticancer Activity In Vitro
Source: Biomolecules. 2022 Oct 21;12(10):1540. doi: 10.3390/biom12101540 (PMC9599051; doi:10.3390/biom12101540)
Supplement: Supplementary file 1 [file biomolecules-12-01540-s001.zip › biomolecules-1962847-supplementary.pdf]

Supplementary Materials

New Betulin Derivatives with Nitrogen Heterocyclic Moiety—Synthesis and Anticancer Activity of *In Vitro*

Ewa Bębenek [1,*], Elwira Chrobak [1,*], Zuzanna Rzepka [2] and Dorota Wrześniok [2]

[1] Department of Organic Chemistry, Faculty of Pharmaceutical Sciences in Sosnowiec, Medical University of Silesia in Katowice, 4 Jagiellońska Str., 41-200 Sosnowiec, Poland

[2] Department of Pharmaceutical Chemistry, Faculty of Pharmaceutical Sciences in Sosnowiec, Medical University of Silesia in Katowice, 4 Jagiellońska Str., 41-200 Sosnowiec, Poland

* Correspondence: ebebenek@sum.edu.pl (EB); echrobak@sum.edu.pl (EC); Tel.: +48-032-364-16-66 (EB); Tel.: +48-032-364-16-69 (EC)

LIST OF CONTENTS

Figure S1. ^{1}H-NMR spectrum for EB366 (600 MHz, $CDCl_3$)

Figure S2. ^{13}C-NMR spectrum for EB366 (150 MHz, $CDCl_3$)

Figure S3. HRMS spectrum for EB366

Figure S4. ^{1}H-NMR spectrum for EB367 (600 MHz, $CDCl_3$)

Figure S5. ^{13}C-NMR spectrum for EB367 (150 MHz, $CDCl_3$)

Figure S6. HRMS spectrum for EB367

Table S1. Cytotoxic *in vitro* activity of indole-functionalized derivatives of betulin, expressed as IC_{50} values.

Table S2. Druglikeness of EB367 predicted using the SwissADME.

Table S3. The *in silico* predictions of human proteins most likely interacting with EB367 computed using SwissTargetPrediction tool.

Table S4. Prediction of the ADME profile of compound EB367 based on computer calculations using admetSAR.

Figure S1. ^{1}H-NMR spectrum for EB366 (600 MHz, $CDCl_3$)

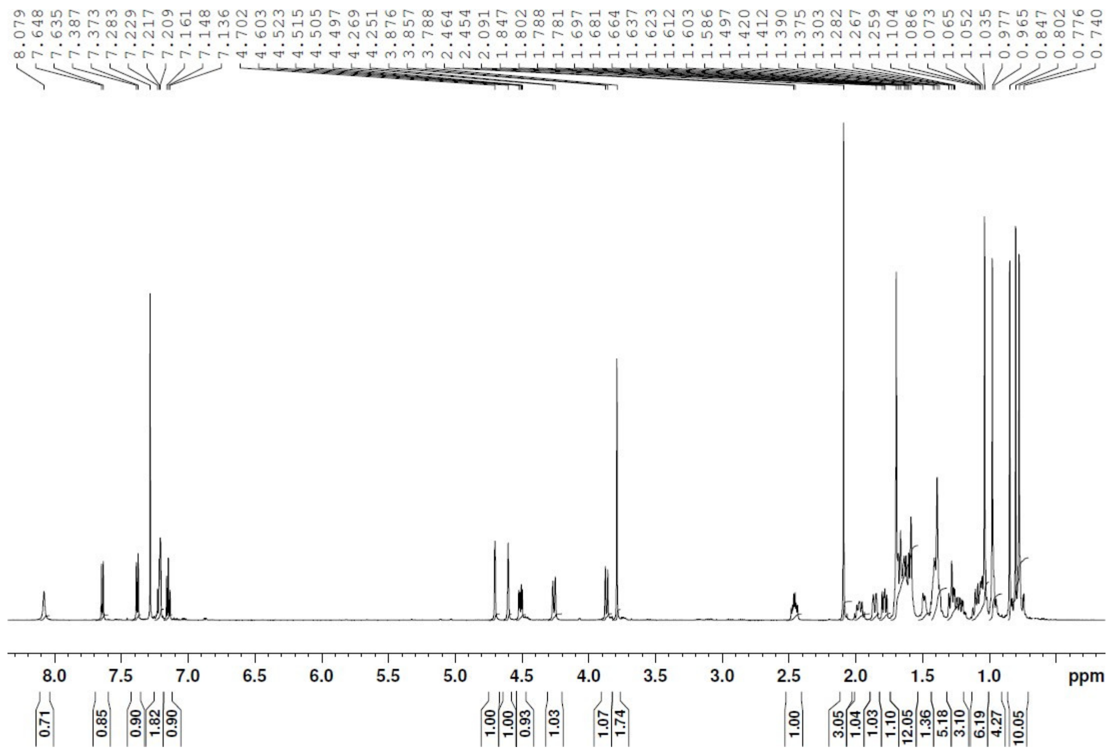

Figure S2. ^{13}C-NMR spectrum for EB366 (150 MHz, $CDCl_3$)

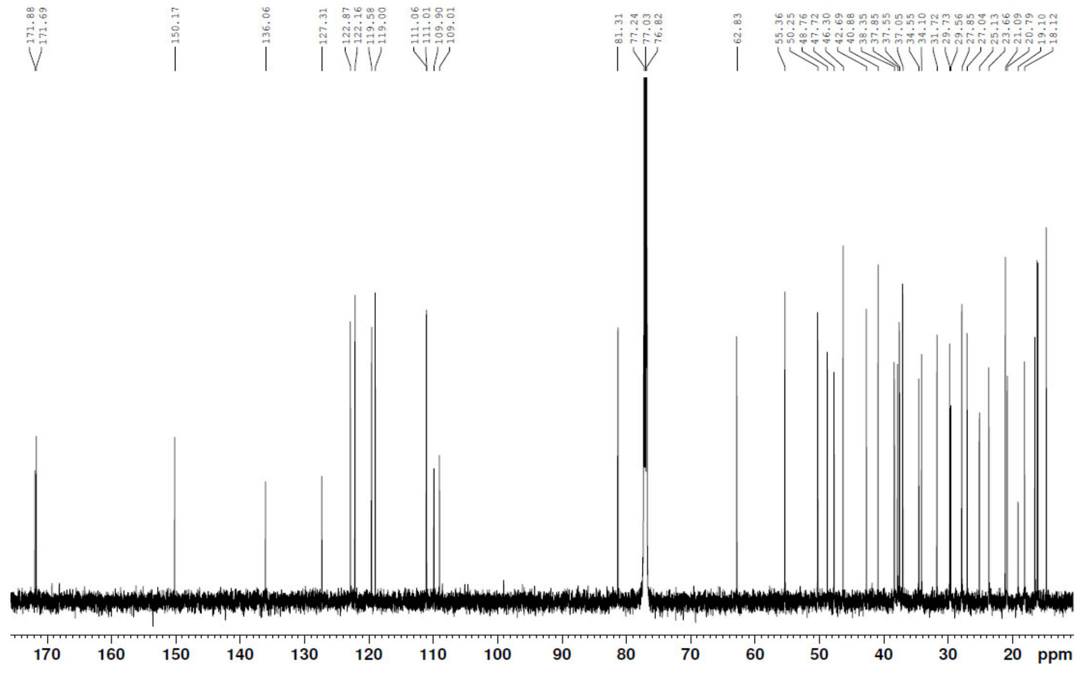

Figure S3. HRMS spectrum for EB366

Analysis Name	D:\Data\EB 366				
Method	APCI_low_ mass_negative.m		Operator	KM	
Sample Name	1-tolil		Instrument	impact II	1825265.10082
Comment					

Acquisition Parameter

Source Type	APCI	Ion Polarity	Negative	Set Nebulizer	2.0 Bar
Focus	Active	Set Capillary	4000 V	Set Dry Heater	200 °C
Scan Begin	100 m/z	Set End Plate Offset	-500 V	Set Dry Gas	4.0 l/min
Scan End	1600 m/z	Set Charging Voltage	2000 V	Set Divert Valve	Source
		Set Corona	15000 nA	Set APCI Heater	450 °C

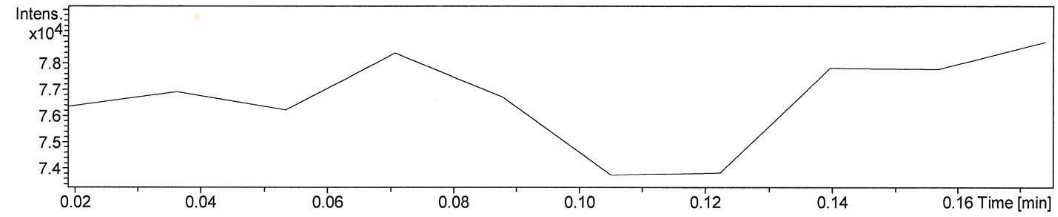

-MS, 0.0-0.1min #2-6

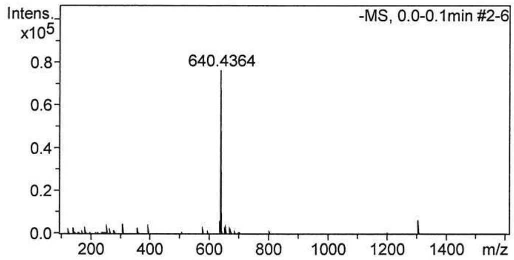

#	m/z	Res.	S/N	I	I %	FWHM
1	640.4364	12004	3626.8	76425	100.0	0.0534

Figure S4. ^{1}H-NMR spectrum for EB367 (600 MHz, $CDCl_3$)

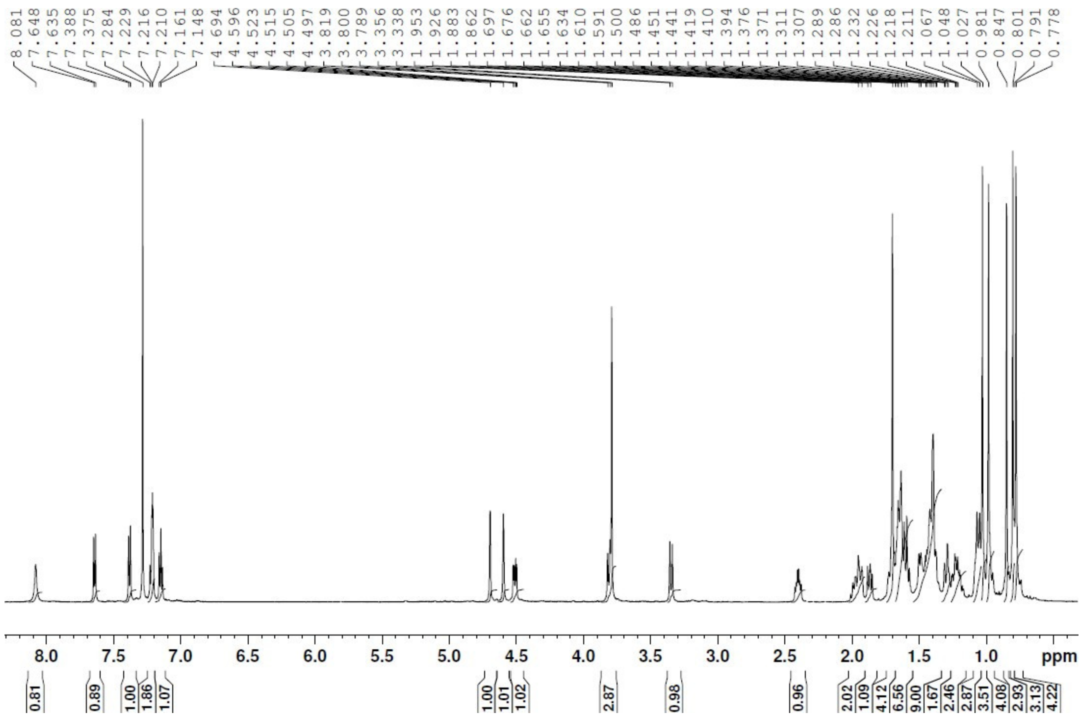

Figure S5. ^{13}C-NMR spectrum for EB367 (150 MHz, $CDCl_3$)

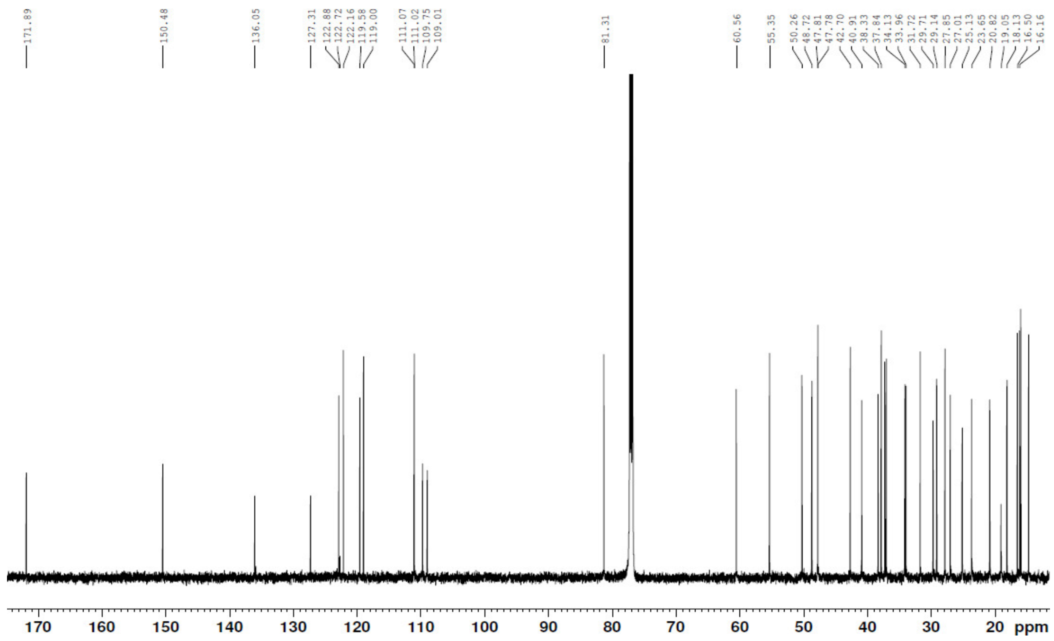

Figure S6. HRMS spectrum for EB367

Analysis Name	D:\Data\EB 367			
Method	APCI_low_ mass_negative.m	Operator	KM	
Sample Name	1-tolil	Instrument	impact II	1825265.10082
Comment				

Acquisition Parameter

Source Type	APCI	Ion Polarity	Negative	Set Nebulizer	2.0 Bar
Focus	Active	Set Capillary	4000 V	Set Dry Heater	200 °C
Scan Begin	100 m/z	Set End Plate Offset	-500 V	Set Dry Gas	4.0 l/min
Scan End	1600 m/z	Set Charging Voltage	2000 V	Set Divert Valve	Source
		Set Corona	15000 nA	Set APCI Heater	450 °C

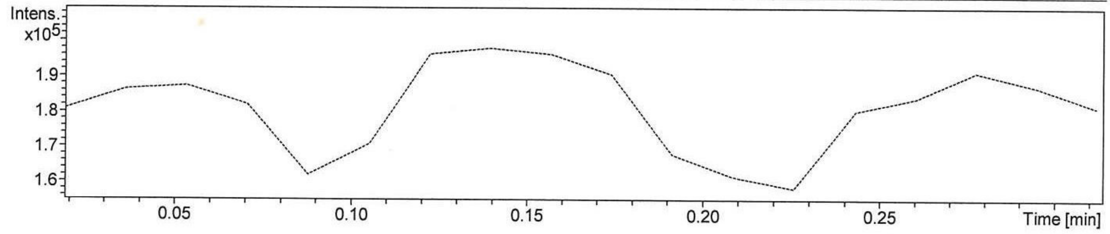

-MS, 0.1-0.1min #3-7

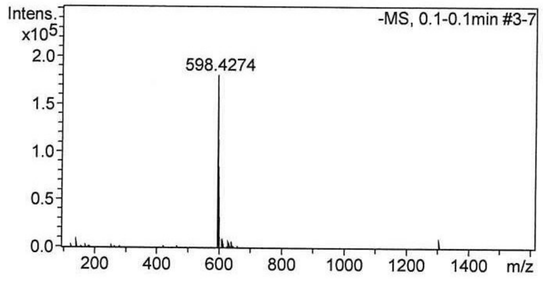

#	m/z	Res.	S/N	I	I %	FWHM
1	598.4274	12267	8529.0	180533	100.0	0.0488

Table S1. Cytotoxic *in vitro* activity of indole-functionalized derivatives of betulin, expressed as IC_{50} values.

	Cell line							
	**NHF**	**A375**	**C32**	**MDA-MB-231**	**MCF-7**	**A549**	**DLD-1**	**HT-29**
**EB366**	Neg	Neg	Neg	Neg	100 µg/ml (167 µM)	Neg	Neg	Neg
**EB367**	Neg	Neg	Neg	Neg	21 µg/ml (35 µM)	Neg	Neg	Neg

Neg – negative in concentrations used; NHF – normal human fibroblasts

Table S2. Druglikeness of EB367 predicted using the SwissADME.

Rule name	Number of violations	Violation
Lipinski	2	MW>500, MLOGP>4.15
Ghose	4	MW>480, WLOGP>5.6, MR>130, #atoms>70
Veber	0	----
Egan	1	WLOGP>5.88
Muegge	1	XLOGP3>5

Ghose rule: 160 ≤MW≤ 480, -04. ≤WLOGP ≤ 5.6, 40 ≤ MR≤ 130, 20 ≤ atoms ≤ 70. 3
Veber rule: rotatable bond ≤ 10, TPSA ≤ 140.
Egan rule: WLOG ≤ 5.88, TPSA≤ 131.6.
Muegge rule: 200 ≤ MW ≤ 600, -2 ≤XLOGP≤ 5, TPSA ≤ 150, num. rings ≤ 7, num. carbon > 4, num. heteroatoms > 1, number of rotatable bonds ≤ 15, H-BA≤ 10, H-BD≤ 5.

Table S3. The *in silico* predictions of human proteins most likely interacting with EB367 computed using SwissTargetPrediction tool.

Target	Common name	Uniport ID	ChEMBL ID	Target class	probability
Cytochrome P450 19A1	CYP19A1	P11511	CHEMBL1978	Cytochrome P450	0.074564954199
Cytochrome P450 17A1	CYP17A1	P05093	CHEMBL3522	Cytochrome P450	0.074564954199
Androgen Receptor	AR	P10275	CHEMBL1871	Nuclear receptor	0.074564954199

Table S4. Prediction of the ADME profile of compound EB367 based on computer calculations using admetSAR.

**Parameter**	Value	Probability
	**Absorption**	
Human intestinal absorption (HIA)	+	0.9874
Caco-2 permeability	-	0.8093
Human oral bioavailability	-	0.6000
	**Distribution**	
Subcellular localization	Mitochondria	0.7201
Blood brain barrier (BBB) permeability	+	0.9407
Organic Anion-Transporting Polypeptide (OATP) inhibitors:		
OATP 2B1	-	0.5688
OATP 1B1	+	0.8642
OATP 1B3	+	0.8896
Multidrug And Toxin Extrusion Transporter 1 (MATE1)	-	0.9212

Organic Cation Transport Protein 2 (OCT2) inhibitor	-	0.5750
Bile Salt Export Pump (BSEP) inhibitor	+	0.9783
P-glycoprotein inhibitor	+	0.7114
P-glycoprotein substrate	+	0.5917
**Metabolism**		
Cytochrome P450:		
CYP450 3A4 substrate	+	0.7439
CYP450 2C9 substrate	-	0.8097
CYP450 2D6 substrate	-	0.8337
CYP450 3A4 inhibition	+	0.7790
CYP450 2C9 inhibition	-	0.5985
CYP450 2C19 inhibition	-	0.5225
CYP450 2D6 inhibition	-	0.8505
CYP450 1A2 inhibition	+	0.5688
CYP inhibitory promiscuity	+	0.8460
